# Stability and Safety of Inhibitor Cystine Knot Peptide, GTx1-15, from the Tarantula Spider *Grammostola rosea*

**DOI:** 10.3390/toxins13090621

**Published:** 2021-09-03

**Authors:** Tadashi Kimura

**Affiliations:** 1 Synthetic Bioengineering Research Group, Bioproduction Research Institute, National Institute of Advanced Industrial Science and Technology (AIST), AIST Tsukuba Central 6, 1-1-1 Higashi, Tsukuba 305-8566, Japan; tadashi.kimura@aist.go.jp; Tel.: +81-29-861-6667; 2 United Graduate School of Drug Discovery and Medical Information Sciences, Gifu University, 1-1 Yanagido, Gifu 501-1193, Japan

**Keywords:** peptide drug, inhibitor cystine knot (ICK), GTx1-15, stability, immunogenicity

## Abstract

Inhibitor cystine knot (ICK) peptides are knotted peptides with three intramolecular disulfide bonds that affect several types of ion channels. Some are proteolytically stable and are promising scaffolds for drug development. GTx1-15 is an ICK peptide that inhibits the voltage-dependent calcium channel Ca_v_3.1 and the voltage-dependent sodium channels Na_v_1.3 and Na_v_1.7. As a model molecule to develop an ICK peptide drug, we investigated several important pharmaceutical characteristics of GTx1-15. The stability of GTx1-15 in rat and human blood plasma was examined, and no GTx1-15 degradation was observed in either rat or human blood plasma for 24 h in vitro. GTx1-15 in blood circulation was detected for several hours after intravenous and intramuscular administration, indicating high stability in plasma. The thermal stability of GTx1-15 as examined by high thermal incubation and protein thermal shift assays indicated that GTx1-15 possesses high heat stability. The cytotoxicity and immunogenicity of GTx1-15 were examined using the human monocytic leukemia cell line THP-1. GTx1-15 showed no cytotoxicity or immunogenicity even at high concentrations. These results indicate that GTx1-15 itself is suitable for peptide drug development and as a peptide library scaffold.

## 1. Introduction

Ion channels play essential roles in a wide range of biological processes (including neural function, muscle contraction and circulation in many physiological systems [1]) and have become attractive pharmaceutical targets related to various conditions such as hypertension, diabetes, pain and cancer [2,3,4,5].

Bioactive peptides from various venomous animals serve as a basis for peptide drug development; examples include ziconotide from cone snails for pain control [6] and exendin-4 from the Gila monster lizard for diabetes treatment [7]. Spider venoms contain various types of bioactive peptides that have attracted attention as peptide drug candidates [8,9,10,11,12,13,14].

**Figure 1:**
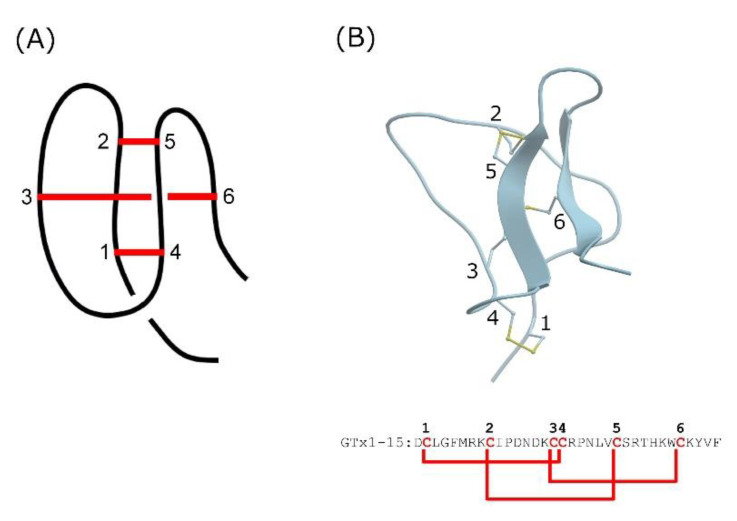
Representative structure of knottin and GTx1-15. (**A**) Cartoon represents peptide backbone and disulfide-bonded cystine-knot core. Red bars indicate disulfide bond connectivity. (**B**) Three-dimensional structure model and amino acid sequence of GTx1-15. 3D structure model of GTx1-15 was constructed via homology modeling with ICM-PRO (Molsoft, La Jolla, CA, USA) based on NMR structures of HnTx-IV (PDB: 1niy). Red letters indicate cysteine residues and red bars indicate disulfide bond connectivity.

Short peptides cannot form structural motifs and are likely to be readily degraded by proteases. Knottins, also known as cystine-knot mini-proteins, are characteristic structural motifs due to multiple covalent bonds formed between side chain thiols of cysteine residues that are spatially distant from each other [15]. Knottins have a compact spherical structure with a very small hydrophobic core due to these disulfide bonds (Figure 1A). The knotted rigid structure is a common feature of knottins and is thought to endow them with unusual proteolytic, thermal and chemical stability [16,17].

Inhibitor cystine knot (ICK) peptides are a class of knottins, which are known to affect several types of ion channel, including voltage-dependent potassium, sodium and calcium channels, as well as mechanosensitive channels [12,18,19]. GTx1-15 is an ICK peptide identified from venom of the tarantula *Grammostola rosea* [20] and thought to have a compact structure due to intramolecular disulfide bonds (Figure 1B). GTx1-15 inhibits both the low-voltage activated calcium channel Ca_v_3.1 [20] and sodium channels Na_v_1.3 and Na_v_1.7 [21].

We previously reported the proteolytic resistance of several ICK peptides such as ProTxI, ProTxII, GsMTx4 and GTx1-15 [22]. GTx1-15 is not degraded by pepsin, trypsin, chymotrypsin and elastase. Based on the proteolytic enzyme resistance of GTx1-15, it is conceivable that GTx1-15 could be used as a template for the development of drugs targeting the gastrointestinal tract. For example, linaclotide is an ICK peptide that targets the adenylate cyclase 2C receptor in the gastrointestinal tract for the treatment of irritable bowel syndrome with constipation [23].

Here, to clarify the eligibility of GTx1-15 as a template for drug development, we report the stability of GTx1-15 in rat and human plasma and its thermal stability. In addition to its stability in vitro, GTx1-15 concentrations in circulating blood after intravenous (i.v.), intramuscular (i.m.) and oral administration were also monitored in vivo. Furthermore, the cytotoxicity and antigenicity of GTx1-15 were determined.

## 2. Results

### 2.1. GTx1-15 Is Not Degraded in Plasma

**Figure 2:**
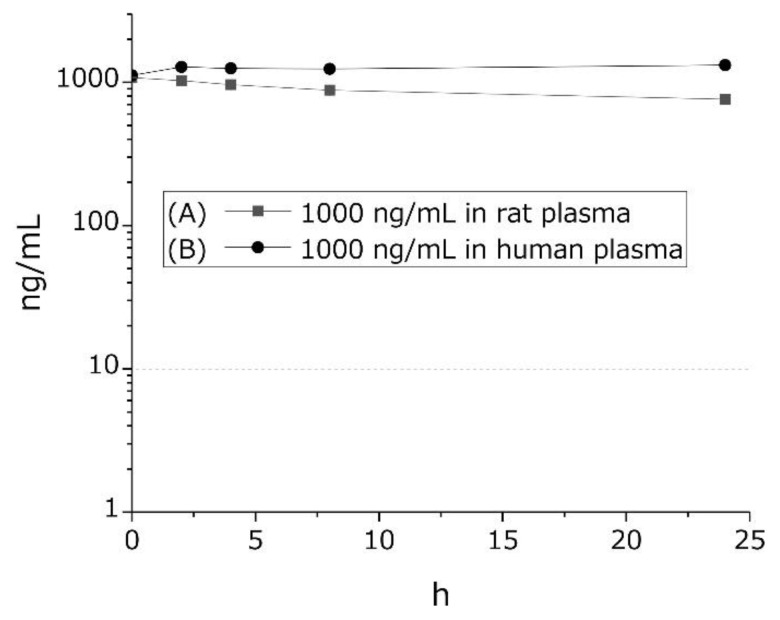
Stability of GTx1-15 in rat or human plasma. GTx1-15 was incubated in rat plasma (**A**) and in human plasma (**B**). No degradation was observed in either rat or human plasma in vitro for 24 h. Results are means ± SEM, *n* = 3. Note that error bars are too small to be visible.

We examined GTx1-15 degradation in rat and human blood plasma. In vitro, 1 μg/mL GTx1-15 was mixed with rat or human blood plasma and incubated at 37 °C for 24 h. In rat plasma, GTx1-15 was only slightly degraded after a 24 h incubation (Figure 2A), while in human plasma, GTx1-15 was not degraded at all after 24 h (Figure 2B).

### 2.2. GTx1-15 Is Stable in Circulation

**Figure 3:**
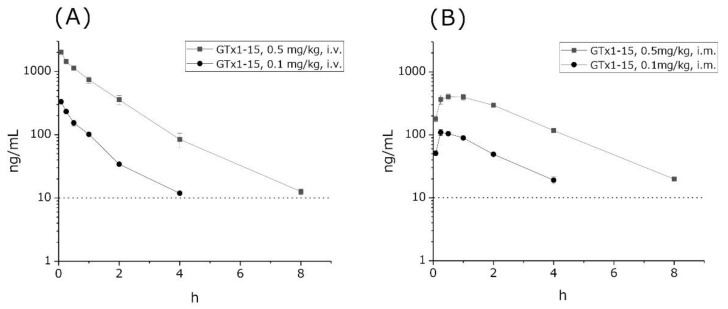
Plasma concentrations of GTx1-15 after i.v. or i.m. administration in rats. (**A**) GTx1-15 concentrations in rat blood after i.v. administration of 0.1 mg/kg or 0.5 mg/kg. GTx1-15 dropped below the detection limit in 4 or 8 h. (**B**) GTx1-15 concentration in rat blood after i.m. administration of 0.1 mg/kg or 0.5 mg/kg. The peak concentration of GTx1-15 in rat blood circulation was detected at 15 min after 0.1 mg/kg administration and at 30 min and 1 h after 0.5 mg/kg administration. Results are means ± SEM, *n* = 3. The dotted line indicates the detection limit of GTx1-15 (10 ng/mL) in blood circulation. No animals administered with GTx1-15 via i.v. or i.m. showed any abnormal behavior throughout the experiments.

The concentrations of GTx1-15 in circulation were investigated using rats. 0.1 or 0.5 mg/kg GTx1-15 was administered to rats in the femoral vein, via i.m. injection, or orally. Then, we observed GTx1-15 concentrations in blood over 24 h. GTx1-15 concentrations in circulation blood gradually decreased and kept dropping to the detection limit (<10.0 ng/mL) dose-dependently within 4 and 8 h after both i.v. (Figure 3A) and i.m. administration (Figure 3B). After peroral administration with 1 or 5 mg/kg of GTx1-15, it was not detected in blood circulation at any time (data not shown). The half-lives of intravenous GTx1-15 in the blood were 30 and 40 min for the 0.1 mg/kg and 0.5 mg/kg doses, respectively. Intramuscular doses of 0.1 mg/kg and 0.5 mg/kg of GTx1-15 showed the highest blood concentrations at 15 and 30 min, respectively, with half-lives of 2 and 3 h.

### 2.3. GTx1-15 Is Stable in High-Temperature Environments

**Figure 4:**
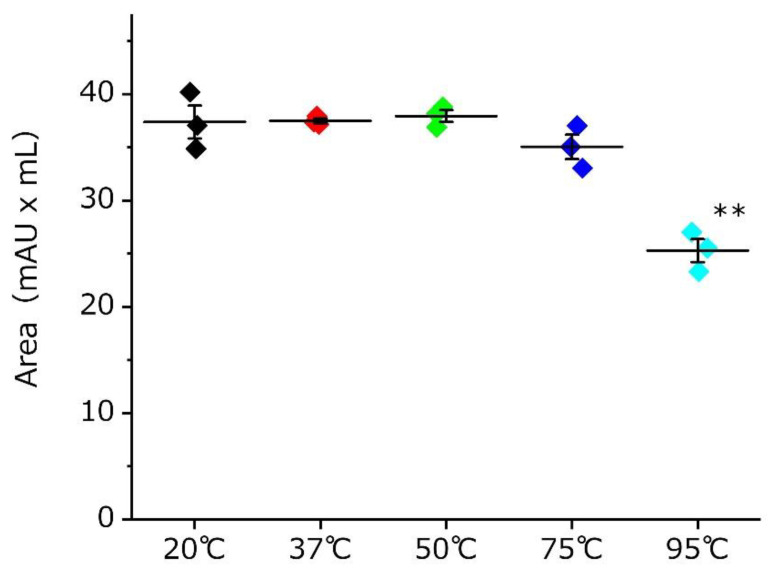
Thermal stability of GTx1-15. The calculated chromatogram peak area after incubation at the indicated temperatures for 24 h are shown. GTx1-15 did not degrade at 20 °C, 37 °C or 50 °C. At 75 °C, GTx1-15 degraded about 5%, but not significantly. About a 30% degradation of GTx1-15 was observed at 95 °C. Experiments were repeated in duplicate, and results are indicated as means ± SEM, *n* = 3. Statistical significance was determined by Dunnett’s multiple test, and *p* values < 0.05 were considered significant. ** indicates a significant difference *p* < 0.01.

Figure 4 indicates that GTx1-15 showed no degradation between 20 °C and 75 °C and was degraded by about 30% with 95 °C incubation for 24 h. Protein thermal shift assays indicated that the Tm B (Boltzmann Tm) values of GTx1-15 and other ICK peptides (ProTxI, ProTxII and GsMTx4) were about 95 °C (Table 1), which means that these ICK peptides show no three-dimensional structural changes in high-temperature environments.

### 2.4. GTx1-15 Shows No Cytotoxicity or Antigenicity

**Figure 5:**
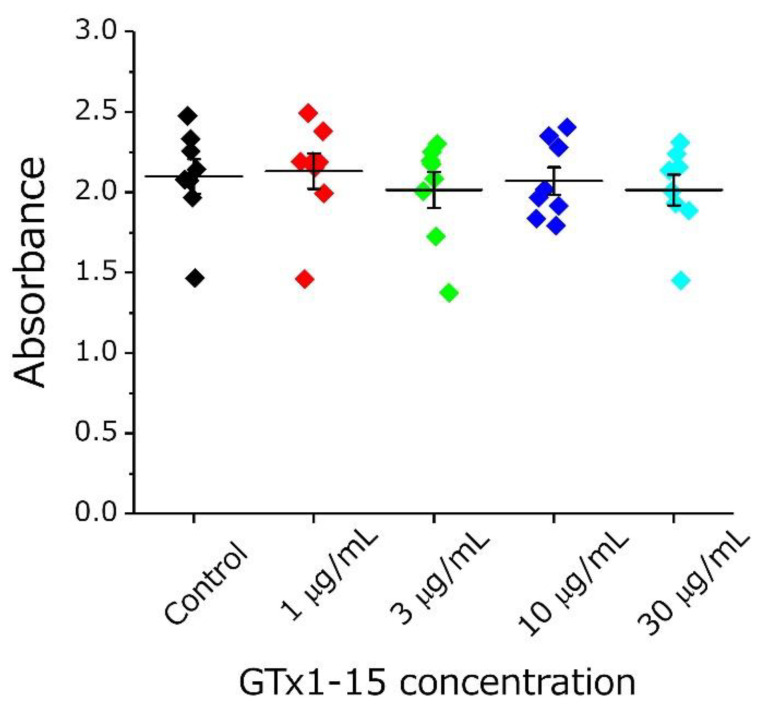
Cytotoxicity of GTx1-15. The effect of GTx1-15 on THP-1 cells after a 24-h exposure is shown. After 2 h of WST-1 incubation, the absorbance at 450 nm was measured by a plate reader. No effect of GTx1-15 was observed. Data are means ± SEM, *n* = 8.

First, the cytotoxicity of GTx1-15 was evaluated using THP-1 cells (a human monocytic leukemia cell line). At all concentrations examined (1, 3, 10 and 30 μg/mL), GTx1-15 had no cytotoxicity (Figure 5).

**Figure 6:**
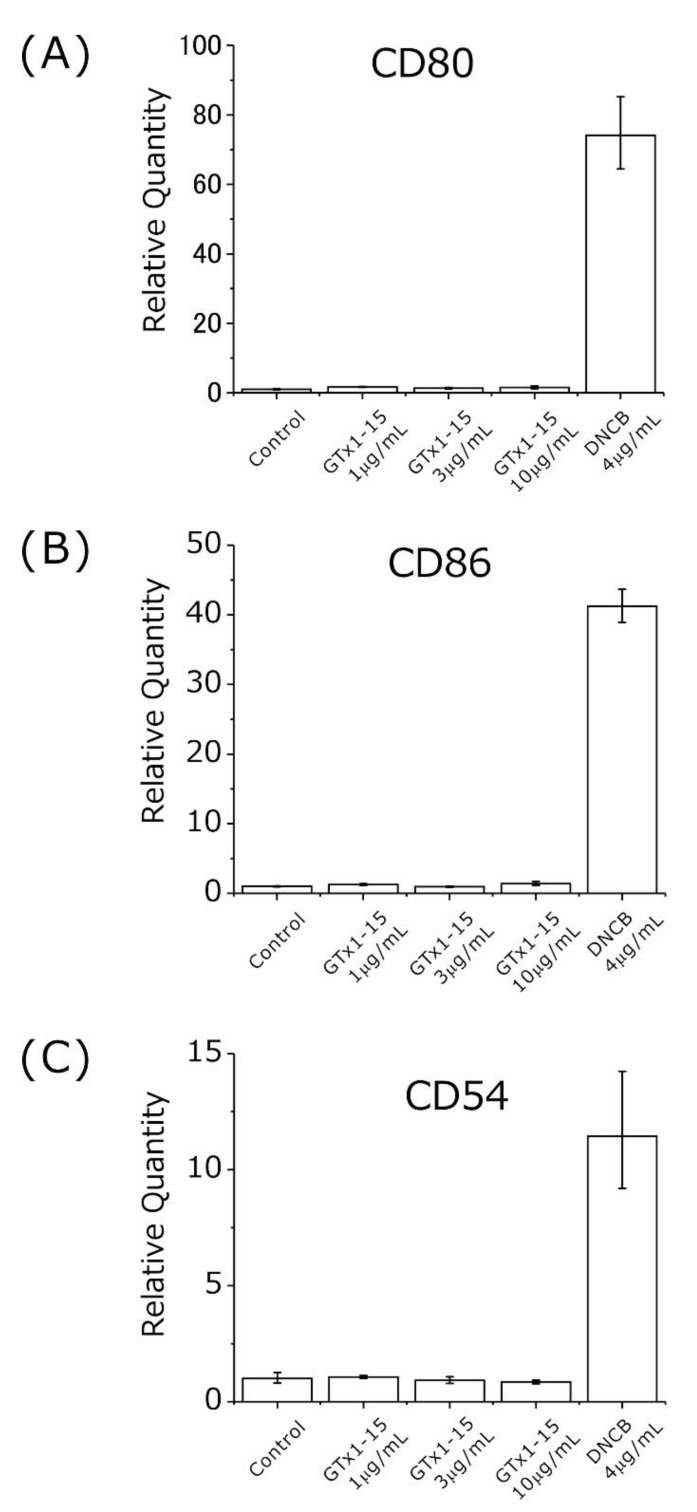
Antigenicity of GTx1-15. The effect of GTx1-15 on THP-1 cells after a 24-h exposure is shown. The expressions of CD80, CD86 and CD54 were quantified by RT-PCR using primers listed in Table 2. No effect of GTx1-15 was observed on CD80 expression (**A**), CD86 expression (**B**), or CD54 expression (**C**). Data are means ± SEM, *n* = 3. Experiments were repeated in triplicate.

Second, the antigenicity of GTx1-15 was evaluated using THP-1 cells at concentrations of 1, 3 and 10 μg/mL. The concentration of 30 μg/mL used in cytotoxicity assays is too high compared with its effective concentration (30 μg/mL: 7.36 μM; Cav3.1 30% inhibition, 40 ng/mL: 9.8 nM (×751); Nav1.3 50% inhibition, 1.2 μg/mL: 300 nM (×25); Nav1.7 50% inhibition, 102 ng/mL: 25 nM (×294)), so the test concentration of 30 μg/mL in the antigenicity assay was omitted. 2,4-Dinitrochlorobenzene (DNCB) is known as a strong allergen and is used in skin sensitization tests. DNCB induces the expression of CD80/CD86 and CD54 when exposed to THP-1 cells [24,25]. Using DNCB as a positive control, approximately 20–70 times higher expressions of CD80, CD86 and CD54 genes were detected after 24 h of exposure (Figure 6). With 1, 3 and 10 μg/mL of GTx1-15, no expression changes of CD80, CD86 and CD54 genes were detected after 24 h of exposure (Figure 6).

## 3. Discussion

### 3.1. The GTx1-15 Molecule Shows High Stability

GTx1-15 concentrations in circulation gradually decreased and kept dropping within 4 and 8 h dose-dependently, meaning that GTx1-15 is stable in blood circulation, which is supported by a previous report showing that GTx1-15 has proteolytic resistance, i.e., GTx1-15 is not degraded by trypsin, chymotrypsin, pepsin or elastase [22].

GTx1-15 inhibits 50% of Na_v_1.3 current at 300 nM and 50% of Na_v_1.7 current at 25 nM [21]. GTx1-15 also inhibits 30% of Ca_v_3.1 current at 9.8 nM in *Xenopus* oocyte two-electrode voltage clamp experiments [20]. Approximately, 9.8 nM of GTx1-15 is equivalent to 40 ng/mL. Four or eight hours after i.v. or i.m. injection, the concentration of GTx1-15 was about 10–20 ng/mL, i.e., 4–8 nM. GTx1-15 might be effective against the target molecules Na_v_1.7 and Ca_v_3.1 for several hours after i.v. and i.m. injection. Since ICK peptides are thought to act by inserting their hydrophobic side into the cell membrane and binding to the ion channel voltage sensor domain on their hydrophilic side [18,26], it is thought that they remain in the cell membranes of the target tissue even after 8 h.

We previously reported that ProTxII (an ICK peptide) remains in circulation for over 4 h [22]. Similar to those results, GTx1-15 retains an effective concentration in circulation for over several hours. Both ProTxII and GTx1-15 are ICK peptides, indicating that ICK peptides might be stable in plasma and are a promising scaffold for peptide drug development.

After peroral administration of GTx1-15, it was not detected in blood circulation at any time. The limit of quantification of GTx1-15 was 10 ng/mL in this experiment. If GTx1-15 absorption from the gastrointestinal tract is less than this limit, then it would not have been measurable by LC-MS/MS. Some ICK peptides have been shown to permeate through rat intestinal mucosa better than other model drugs [27]. It might be that GTx1-15 is absorbed from the gastrointestinal tract at very low concentrations that are under the limit of quantification. To detect very low concentrations of GTx1-15 in circulation after peroral administration, more sensitive peptide detection methods, such as the use of radio-iodinated GTx1-5, are required. Using iodinated GTx1-15, it would be possible to show the absorption of GTx1-15 from the intestinal tract and its permeability to the brain. Ziconotide (Prialt), which is an ICK peptide from cone snails and is already on the market, is known to transfer to the brain [28]. On the other hand, it is possible that GTX1-15 is not absorbed from the intestinal tract at all. In that case, GTx1-15 and its derivatives could be developed as drugs that target molecules present in the intestinal tract such as linaclotide [23] or used as lead compounds for drugs with improved absorption from the intestinal tract. Modification of GTx1-15 with reference to chlorotoxin, which is used for targeting glioblastoma [29], and crotamine, a cell membrane-permeable peptide [30], may improve intestinal absorption.

**Figure 7:**
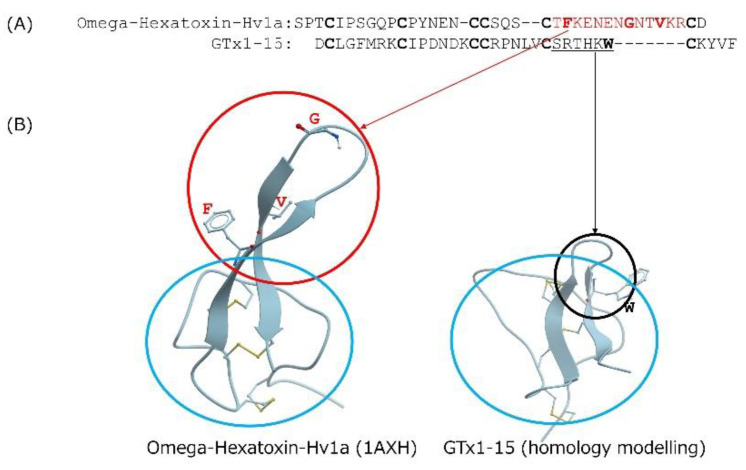
Comparison of GTx1-15 and ω-hexatoxin-Hv1a. (**A**) Amino acid comparison of GTx1-15 and ω-hexatoxin-Hv1a. Cysteine residues are indicated in bold letters. Note that ω-hexatoxin-Hv1a contains a long loop in the C-terminal region of the molecule (amino acid residues shown in red). Hydrophobic amino acid residues are shown in red bold letters. (**B**) 3D structure comparison of GTx1-15 and ω-hexatoxin-Hv1a. 3D structure models of GTx1-15 was constructed by homology modeling with ICM-PRO (Molsoft, La Jolla, CA) based on NMR structures of HnTx-IV (PDB: 1niy). 3D structure of ω-hexatoxin-Hv1a is based on PDB No. 1AXH. The long loop part of ω-hexatoxin-Hv1a (the area circled in red) protrudes from the main body consisting of three disulfide bonds (the area circled in blue). However, the very small loop part of GTx1-15 (the area circled in black) is different from the ω-hexatoxin-Hv1a long loop. The hydrophobic amino acid residues shown in red bold in (**A**) are indicated by a single letter at the corresponding position on the ribbon. All hydrophobic amino acid residues are located outside of the blue circle. However, tryptophan, a hydrophobic amino acid residue shown as a bold letter in the small loop of GTx1-15, is located inside the blue loop.

Cyclotides are cystine-knotted peptides in which the N- and C-termini are cyclized. Kalata B1 is a well-known cyclotide that consists of 29 amino acids and is not degraded after heating to temperatures approaching boiling [15]. The insecticidal spider toxin ω-hexatoxin-Hv1a is an ICK peptide whose thermal stability has been investigated [15]. After incubating it for 24 h at 50 °C, 75 °C or 95 °C, ω-hexatoxin-Hv1a is degraded about 20%, 30% and 90%, respectively. In contrast, GTx1-15 degraded only 30% after a 24-h incubation at 95 °C. GTx1-15 thus shows higher thermal stability than ω-hexatoxin-Hv1a. In comparing the amino acid sequences of GTx1-15 and ω-hexatoxin-Hv1a (Figure 7A), the c-terminal region of ω-hexatoxin-Hv1a has a long loop region compared to GTx1-15 (Figure 7B). The long loop of ω-hexatoxin-Hv1a protrudes from the main body consisting of three disulfide bonds, which is different from the compact structure of GTx1-15, and this protrusion is thought to cause instability at high temperatures by forming aggregates. This might be why ω-hexatoxin-Hv1a is more unstable than GTx1-15 at high temperatures.

### 3.2. GTx1-15 Is Safe with No Cytotoxicity or Antigenicity

It widely accepted that little or no cytotoxicity or antigenicity of peptide drugs is necessary for their safe use. First, we evaluated the cytotoxicity of GTx1-15 using THP-1 cells (a human monocytic leukemia cell line) and a WST-1 cell proliferation assay kit. We quantified the cell proliferation activity and viability to evaluate the cytotoxicity. As a result, GTx1-15 showed no cytotoxicity at any concentration tested.

Second, to clarify whether GTx1-15 is immunogenic, its antigenicity was evaluated using THP-1 cells at concentrations of 1, 3 and 10 μg/mL by detecting CD80, CD86 and CD54 expression using RT-PCR quantification. CD80 and CD86 are expressed in dendritic cells, monocytes and antigen-presenting cells such as macrophages. These molecules bind with CD28 or CD152 expressed in T cells in the case of antigen presentation and participate in the activation or inhibition of T cells as a costimulatory pathway [31]. In contrast, although CD54 binds with CD11/CD18 of T cells and contributes to the costimulatory pathway, CD54 is expresses in various cells such as vascular endothelial cells as well as antigen-presenting cells. These molecules work as adhesion factors and are important in lymphocyte migration [32]. Among these molecules, CD86 and CD54 are used as molecular markers for antigenicity tests [25,25,33]. A GTx1-15 concentration of 30 μg/mL is equivalent to 7.5 μM, and this concentration is about 750 times higher than that (9.8 nM) at which GTx1-15 inhibits 30% of calcium currents expressed in *Xenopus* oocytes. So, the concentration of 30 μg/mL was omitted from antigenicity assay. In the antigenicity assays, a 24-h exposure to DNCB, as a positive control, induced approximately 20–70 times higher expression of CD80, CD86 and CD54 genes. With 1, 3 and 10 μg/mL of GTx1-15, no expression changes in CD80, CD86 and CD54 genes were detected after 24 h of exposure. These results indicate that antigenicity was not detected after 24 h of exposure with concentrations of GTx1-15 that are 25, 75 and 250 times higher than the 30% inhibition concentration of about 9.8 nM.

Taken together, GTx1-15 will likely have little or no antigenicity if injected systemically in humans, especially for a short-term period, implying that it may be safe to develop GTx1-15 as a pharmaceutical product. To clarify this point, larger-scale experiments using a wide range of healthy cell lines from various body tissues would be necessary.

### 3.3. GTx1-15 Is a Suitable Scaffold for Peptide Library Construction

Recently, we developed an *Escherichia coli* periplasmic peptide display technique, PERISS (intra periplasm secretion and selection). In the PERISS method, a target protein (we focused on ion channels) and a peptide library (we focused on an ICK peptide library) are coexpressed in the *E. coli* inner-membrane and periplasmic space, respectively [34].

It is assumed that membrane proteins that are expressed in the inner membrane have correct three-dimensional structures and correct activity. To verify this assumption, we have developed an *E. coli* giant spheroplast electrophysiological technique [35]. Using this technique, we can measure induced potassium channel currents. Peptide library screening against correctly expressed ion channels should be able to identify new bioactive peptides.

The *E. coli* periplasmic space is suitable for the expression of disulfide-rich peptides [34,36,37,38,39], and the ICK peptide scaffold is suitable for directed molecular evolution to generate new peptide drugs [40,41,42,43]. The current study revealed that GTx1-15 possesses high stability (thermal stability and stability in circulation) and is safe at the cellular level (no cytotoxicity or antigenicity). Therefore, peptides screened from a GTx1-15-based peptide library might have the same characteristics of high stability and safety in vivo. We have prepared a peptide library using GTx1-15 as a scaffold to screen with the PERISS method, and after screening with PERISS against an ion channel, several bioactive peptides were successfully obtained [44].

## 4. Conclusions

ICK peptides are viable drug leads based on their high target specificity, high affinity, and thermal, chemical and proteolytic stability. Our results show that little or no degradation of GTx1-15 occurred in rat or human plasma for 24 h in vitro. GTx1-15 concentrations in circulation gradually decreased and kept dropping within 4 and 8 h, implying that the main clearance pathway might be the renal or other pathways (but not degradation) and pharmacologically effective peptide concentrations remain for several hours compared with other nonmodified peptides. GTx1-15 has no cytotoxicity and no antigenicity as revealed by THP-1 cell exposure tests. GTx1-15 has potential as a lead for a novel class of peptide drugs that are highly stable with low cytotoxicity and antigenicity. Finally, GTx1-15 is suitable as a template in a peptide library for periplasmic peptide display.

## 5. Materials and Methods

### 5.1. Peptides and Chemicals

GTx1-15 was obtained from Alomone Labs (Jerusalem, Israel). Human plasma was purchased from KOJIN BIO (Saitama, Japan). The protein thermal shift starter kit was from Thermo Fisher Scientific (Tokyo, Japan). RPMI-1640 with L-glutamine and phenol red, sodium hydrogen carbonate, and Tris-HCl were purchased from Wako Chemicals (Osaka, Japan). A human monocytic leukemia cell line, THP-1 (No. JCRB0112), was from the Japanese Collection of Research Bioresources Cell Bank (Osaka, Japan). Penicillin-streptomycin solution was obtained from Life Technologies Japan Ltd. (Tokyo, Japan). HyClone fetal bovine serum was purchased from GE Healthcare Japan (Tokyo, Japan). Premix WST-1 reagent was purchased from Takara Bio (Shiga, Japan).

### 5.2. Stability Assays in Animal Plasma In Vitro

To observe peptide stability in vitro, blood was extracted from three 8-week-old male SD rats after anesthesia with isoflurane inhalation, and the plasma was separated by centrifugation at 1850× *g* for 10 min at 4 °C. Plasma was stored on ice and used within the day of the experiment. Human plasma was stored at −80 °C until use after purchase. GTx1-15 was added to the plasma to make a 1 µg/mL mixture and incubated at 37 °C for 24 h, withdrawing 250 µL aliquots at 0, 2, 4, 8 and 24 h. Samples were kept at −25 °C away from light until analysis. This experiment, including LC-MS/MS sample analysis, was conducted by Nemoto Science Co., Ltd. (Ibaraki, Japan).

### 5.3. Concentration in Circulation after Intravenous, Intramuscular, and Peroral Administration

To observe GTx1-15 concentrations in blood circulation in vivo, three nonfasted male rats were administered with 0.1 or 0.5 mg/mL/kg of GTx1-15 i.v. or i.m. under isoflurane anesthesia, or peroral administration with 1 or 5 mg/mL/kg of GTx1-15 without any anesthesia. Blood samples of 450 μL were taken from the tail veins at 0.083, 0.25, 0.5, 1, 2, 4, 8 and 24 h after administration using Pasteur pipettes coated with heparin sodium. Plasma was obtained via centrifugation at 10,000× *g* for 3 min at 4 °C and stored at −25 °C protected from light until analysis. Animal experiments, including LC-MS/MS analysis, were conducted by Nemoto Science Co., Ltd. in accordance with the guidelines of the animal experiment ethics committee (authorization numbers: 2016-0012, 2016-0073, 2017-0121) and under approval of the animal experiment ethics committee of National Institute of Advanced Science and Technology (authorization numbers: A2015-119, A2016-199, A2017-199).

### 5.4. LC-MS/MS

For LC-MS/MS sample preparation, 100 µL plasma was mixed with 20 µL 50% methanol and 200 µL 4% phosphoric acid. Whole sample mixtures were added to an Oasis HLB 1 cc/10 mg extraction cartridge (Waters, MA, USA) equilibrated with 1 mL methanol and 1 mL distilled water. The column was washed with 1 mL 5% methanol and eluted with 1 mL methanol. The eluate was dried under nitrogen flow and dissolved in 100 µL solvent A/B (30%:70%, *v*/*v*) for LC-MS/MS. 10 µL samples were analyzed by a Waters LC-MS/MS unit. ACQUITY UPLC BEH HILIC, 1.7 µm, 2.1 mm I.D. × 100 mm (Milford, MA, USA), was used at a flow rate of 0.3 mL/min by linear gradient elution (solvent A: solvent B = 30%:70%, *v*/*v*; solvent A: 0.1% TFA, solvent B: acetonitrile) using electro-spray ionization for Xevo TQ MS (Waters).

### 5.5. Thermal Stability

Thermal stability was examined by incubating GTx1-15 (24.7 µM, 10 μg/100 μL) for 24 h at various temperatures (20, 37, 50, 75 and 95 °C). After 24 h, samples were immediately stored at −20 °C prior to HPLC fractionation. Each 100 μL sample was diluted with 400 μL 0.05% TFA solution and subsequently separated using a Superiorex ODS column (4.6 × 250 mm, Shiseido) eluted with a linear gradient of 0–30% acetonitrile containing 0.05% TFA at a flow rate of 1 mL/min using AKTA pure25 (GE Healthcare). The peak area measured at wavelength 214 nm, which represents the native GTx1-15 quantity, was calculated using the evaluation function of Unicorn software version 7.1.

### 5.6. Protein Thermal Shift Assay

The protein thermal shift assay was conducted with the StepOne Real-Time PCR System (Thermo Fisher Scientific) using the Protein Thermal Shift Assay Kit according to the manufacturer’s instructions with Protein Thermal Shift Software version 1. The protein melt reaction mix was added to the wells of a 48-well PCR plate. The plate was heated from 25 to 99 °C with a heating rate of 1 °C/min. The software allows the user to calculate a melting temperature (Tm) from their melt curve data using the Boltzmann equation (Tm B).

### 5.7. Cytotoxicity Assay

THP-1 cells were cultured in RPMI-1640 containing 10% fetal bovine serum and supplemented with L-glutamine and penicillin/streptomycin. Cells were grown in a humidified 5% CO_2_ incubator at 37 °C. For cytotoxicity assays, 3 × 10^4^ THP-1 cells were inoculated into single wells of a 96-well plate with 80 μL RPMI-1640 medium. Immediately after inoculation, 20 μL of GTx1-15 solution were added to the wells. Final concentrations of GTx1-15 were 1, 3, 10 and 30 μg/mL. As a control, RPMI-1640 medium was added to the wells instead of GTx1-15 solution. After a 24-h incubation, cytotoxicity was tested according to manufacturer’s instructions. Briefly, 10 μL WST-1 solution was added into each well and incubated for 2 h at 37 °C. After incubation, the absorbance was measured using a microplate reader (Eppendorf, Hamburg, Germany) at 450 nm.

### 5.8. Antigenicity Assay

THP-1 cells were cultured as described above. For antigenicity assays, 2.4 × 10^6^ THP-1 cells were inoculated into single wells of a 12-well plate with 1.2 mL RPMI-1640 medium. Immediately after inoculation, GTx1-15 solution or DNCB solution were added to each well. Final concentrations of GTx1-15 were 1, 3 and 10 μg/mL, and the DNCB concentration was 4 μg/mL as a positive control. As a negative control, RPMI-1640 was added. After a 24-h incubation, THP-1 cells were collected via centrifugation at 1000× *g* for 5 min, and then total RNA was extracted from cells using an RNeasy Mini Kit (QIAGEN, Hilden, Germany) according to the manufacturer’s instructions. Total RNA was treated with DNase (QIAGEN) for 10 min to digest contaminating genomic DNA and purified by an RNeasy MinElute Cleanup Kit (QIAGEN). Complementary DNA was synthesized with PrimeScript Reverse Transcriptase (Takara Bio) with an oligo (dT)12-18 primer (Invitrogen, Waltham, USA), 10 mM each dNTP mixture (Promega, Madison, USA), and RNase Inhibitor (Takara Bio). Real-time PCR mixtures were prepared with SYBR Premix Ex Taq (Takara Bio) according to the manufacturer’s instructions. The reaction and monitoring were performed with a StepOne Real-Time System (Thermo Fisher Scientific) for 40 cycles of two-step shuttle PCR (95 °C for 5 s, 60 °C for 30 s). Primers for real-time PCR were designed using Roche ProbeFinder version 2.45 (http://qpcr.probefinder.com/roche3.html. This site is stopped at the end of 2020). Primers used for the detection of human CD80, CD86, CD54 and GAPDH are shown in Table 2.

## Figures and Tables

**Table 1:** Protein thermal shift assay.

ICK Peptide	Tm B (°C)
GTx1-15	96.30 ± 0.03
ProTxI	93.23 ± 0.12
ProTxII	93.95 ± 0.12
GsMTx4	95.26 ± 0.10

Tm B: Boltzmann melting temperature.

**Table 2:** Primers used to detect human CD80, CD86, CD54, and GAPDH in real-time PCR.

Gene		Sequence	Amplicon Size (bp)
CD80	Forward Reverse	CCTACTGCTTTGCCCCAAGA AAGGGCAAGGTGGGGTAATC	188
CD86	Forward Reverse	ACGCGGCTTTTATCTTCACC TCTTCCCTCTCCATTGTGTTGG	200
CD54	Forward Reverse	TTGAGGGCACCTACCTCTGT GATCTTCCGCTGGCGGTTAT	176
GAPDH	Forward Reverse	CCATGGAGAAGGCTGGGG CAAAGTTGTCATGGATGACC	195

CD80: Cluster of differentiation 80, CD86: Cluster of differentiation 86, CD54: Cluster of Differentiation 54, GAPDH: Glyceraldehyde-3-Phosphate Dehydrogenase, bp: base pair.

## Data Availability

The data presented in this study are available upon request to the corresponding author.
